# Effectiveness and feasibility of lowering playground density during recess to promote physical activity and decrease sedentary time at primary school

**DOI:** 10.1186/1471-2458-13-1154

**Published:** 2013-12-10

**Authors:** Sara D’Haese, Delfien Van Dyck, Ilse De Bourdeaudhuij, Greet Cardon

**Affiliations:** 1 Faculty of Medicine and Health Sciences, Department of Movement and Sports Sciences, Ghent University, Watersportlaan 2, Ghent 9000, Belgium; 2 Research Foundation Flanders (FWO), Egmontstraat 5, Brussels 1000, Belgium

**Keywords:** Intervention, Youth, Physical activity, School, Playground, Sedentary behavior

## Abstract

**Background:**

This pilot study aimed at investigating the effectiveness of lowering playground density on increasing children’s physical activity and decreasing sedentary time. Also the feasibility of this intervention was tested.

**Methods:**

Data were collected in September and October 2012 in three Belgian schools in 187, 9–12 year old children. During the intervention, playground density was decreased by splitting up recesses and decreasing the number of children sharing the playground. A within-subject design was used. Children wore accelerometers during the study week. Three-level (class – participant - measurement (baseline or intervention)) linear regression models were used to determine intervention effects. After the intervention week the school principals filled out a questionnaire concerning the feasibility of the intervention.

**Results:**

The available play space was 12.18 ± 4.19 m^2^/child at baseline and increased to 24.24 ± 8.51 m^2^/child during intervention. During the intervention sedentary time decreased (−0.58 min/recess; -3.21%/recess) and moderate-to-vigorous physical activity (+1.04 min/recess; +5.9%/recess) increased during recess and during the entire school day (sedentary time: -3.29%/school day; moderate-to-vigorous physical activity +1.16%/school day). All principals agreed that children enjoyed the intervention; but some difficulties were reported.

**Conclusions:**

Lowering playground density can be an effective intervention for decreasing children’s sedentary time and increasing their physical activity levels during recess; especially in least active children.

## Background

As physical activity during childhood is associated with numerous health benefits [1-3], social well-being [4,5] and academic achievement [6], it is recommended for children to engage in at least 60 minutes of moderate-to vigorous-intensity physical activity (MVPA) per day [7,8]. Independent of the amount of physical activity, high levels of sedentary time (sitting and lying activities that do not increase energy expenditure substantially above the resting level [9]) are associated with obesity and metabolic risks [10,11]. Therefore, the amount of sedentary time should be limited [12]. Despite numerous health benefits associated with being sufficiently active and limiting sedentary time, many children do not meet the physical activity guidelines (e.g. only 4.6% of the girls and 16.8% of the boys in Europe meet MVPA guidelines with 1.7% of the Belgian girls and 14.0% of the Belgian boys meeting the guidelines) and spend too much time being sedentary [13].

Physical activity in children declines as children age [14-16], and therefore, it is important to promote physical activity during childhood. As most children spend a large proportion of their waking hours at school, elementary school is an ideal setting to promote physical activity. Moreover, children from different ethnic and socio-economic groups can be reached in elementary schools [17]. During a school day, recess and physical education offer opportunities to contribute to the recommended 60 minutes of MVPA per day [18]. Moreover, recess takes place on a daily basis and accounts for nearly a quarter of the average school day in Flanders. Nonetheless, children’s physical activity levels during recess are low, especially in girls [19]. Ridgers et al. hypothesized that children should engage in MVPA for a minimum of 40% of recess; however, only 14.9% of the boys and 4.3% of the girls met this recommendation in the UK [19]. These findings show that increasing children’s MVPA during recess is an essential and realistic objective and strategic interventions are needed to increase physical activity levels of children during recess [19,20].

Recently, two reviews studied the interventions that have been trialed to promote physical activity in recess settings [21,22]. Different intervention strategies were identified. Most of the interventions added equipment or materials to the regular playground offerings and also playground markings were frequently used. Furthermore teacher involvement, physical structures (e.g. soccer goal posts, basketball hoops), color coded playground areas and zones, court rotation, organized activities and active video games in order to increase children’s physical activity levels during recess were investigated. Most of these interventions were successful in increasing children’s physical activity levels during recess [21,22]. While in a recent focus group study, lack of access to space was recognized as an important barrier for active play during recess according to 10- to 13-year old children [23], the effect of more play space for children during recess on their physical activity has not been studied yet.

In a cross-sectional study, available play space per child was a significant negative predictor of observed sedentary time and a positive predictor of observed vigorous activity [24] and Harten et al. found in an explorative study that boys (mean age = 9.3 years ± 0.5) in large-space areas were more objectively active compared to boys in small-space areas [25]. In a subsample of the latter study, the effect of increasing playground density by using markers to decrease play space on children’s estimated energy expenditure, based on activity counts measured by activity monitors, was also investigated. By decreasing playground space, it was found that boys were less active in smaller play areas (13.0 - 15.8 m^2^ per child) compared to medium (71.6 - 86.8 m^2^) and large (130.4 – 157.9 m^2^) play areas [25].

It is not obvious to put more play space at children’s disposal during recess, as many schools do not have enough space to increase the playground area. A possible solution for this problem is to decrease playground density by splitting up recesses and consequently decreasing the number of children sharing the playground. Not only children’s play space will increase, but children will also have better access to sports materials (e.g. soccer and basketball hoops, playground markings, loose materials,..) as fewer children are having recess at the same time. Moreover, such an intervention is free of cost and does not require any additional training of supervisors.

In preschool children, lowering playground density, by splitting up recesses, had a positive effect on preschoolers’ objectively measured physical activity levels and was effective in lowering sedentary time [26]. However no studies were found investigating the effectiveness of lowering playground density during recess in elementary schools.

Therefore, the primary aim of this pilot study was to test the effectiveness of lowering playground density in order to increase physical activity levels and to decrease sedentary time in 9–12 year old-children. Harten et al. concluded that play space was an important variable in determining the free play activity of boys, but not of girls [25]. Therefore it is expected that this intervention might affect boys and girls differently and the moderating effect of sex in the relation between play space and children’s physical activity was tested.

More active children are more autonomously motivated [27] and are more likely to be active, independently of the available play space. Consequently it is hypothesized that less active children will benefit more from this intervention compared to more active children. Therefore, moderating effects of children’s baseline physical activity during recess were tested. Furthermore Fremeaux et al. stated that more activity at one time (e.g. during the intervention recess) will be compensated for by less activity at another time (e.g. during another recess) [28] due to the effect of intrinsic biological control on physical activity [29]. This implies that the decrease of playground density might be successful during recess, but cannot be detected in a measure of physical activity during the whole school day [28]. Therefore, the intervention effects on children’s physical activity and sedentary behavior during the entire school day were also tested. By doing so, the activitystat hypothesis, was tested [28]. The term ‘activitystat’, refers to the central control of PA according to a set point for energy expenditure. As the actual implementers of the intervention were the teachers, and not the researchers it is important to investigate if the intervention was attainable to conduct in order to increase sustainability. Therefore, a third aim of this study was to test the feasibility of lowering playground density during recess in primary school. Additionally, cross-sectional baseline associations between play space and physical activity were investigated.

## Methods

### Participants

Data of the present study were collected in September and October 2012 in 3 Belgian schools during one week per school (Monday until Friday). In total, 187 children aged 9–12 years participated in the study; 53 children (28.3%) from school 1; 69 children (36.9%) from school 2 and 65 children (34.8%) from school 3.

In total, a convenience sample of 10 schools was selected from elementary schools in the Province Flemish Brabant and contacted by phone. In two of these schools the playtime had been split up already due to lack of space, in another school classes were located around the playground and splitting up playtime would cause nuisance for the surrounding classes, three principals thought it would be too difficult to schedule the recesses, and one school thought that it would be too difficult to find enough supervisors during the split recesses. In total, 3 principals agreed to participate in the intervention and feasibility study (response rate schools = 30.0%).

In each school, all teachers of the 4th, 5th and 6th grade were asked to let their class participate in the study and 10/12 (83.3%) teachers agreed to participate. In each class, all children were invited to participate in the study.

The parents of 187 children from the fourth, fifth and sixth grade, gave informed consent to participate in the intervention study (response rate = 88,2%). The Ethics Committee of the Ghent University Hospital approved the study.

### Procedure

A within subject design was used to test the effectiveness of lowering playground density during recess in this pilot study. School principals were asked to schedule two days with usual recesses and three days with recesses with lowered playground density during the study week. Principals were free to choose on which days the intervention took place and during which recess (before noon, during lunch or after noon) and they had to provide a supervisor during the extra recess. One intervention recess was scheduled per day and children received all other recesses as usual. All principals chose to implement the intervention before noon (1 principal) or during the afternoon recesses (2 principals). During recesses in the usual conditions (baseline), all classes had recess at the same time (e.g. from 10h00 until 10h15). During a recess with lowered playground density (intervention) the number of classes usually sharing the playground was divided into two (e.g. grades 1, 2 and 3 had recess from 9h45 until 10h00; grades 4, 5, 6 had recess from 10h00 until 10h15). All recesses (intervention and non-intervention) were supervised by classroom teachers. Within one week after the intervention, the school principals were asked to fill in a questionnaire about the feasibility of implementing the intervention.

### Measurements

Date of birth and sex of the participants were obtained from the school principals. Recess times and school times were obtained from the class teachers from each class.

### Playspace per child

Google™ Earth software was used to calculate the area of the playground in each school; using aerial pictures of the playgrounds and the polygon measurement tool. This software has been used previously to compute the area of playgrounds [24].

The number of children at the playground during each recess was obtained from the class teachers during daily researcher visits. By dividing the playground area by the number of children in the school, the available play space per child was calculated.

### Physical activity and sedentary behavior

Each school was visited by a researcher on Monday morning (before the first recess) and Friday afternoon (after the last recess) of the study week, to distribute and collect the accelerometers. Participants wore an Actigraph GT3X + accelerometer (15 s epoch [30-33]) during waking hours from Monday until Friday. The accelerometer was worn on the right hip secured by an elastic waist belt. The researcher demonstrated how to wear the accelerometer in the classroom. Children were asked to wear the accelerometer during waking hours but to remove it for aquatic activities (e.g. bathing, swimming). Accelerometer data were screened, cleaned and scored using data-reduction software MeterPlus 4.2. Periods of 60 minutes of consecutive zeros or more were removed and defined as non-wearing time [34-36]. Accelerometer data were scored using the cutpoints of Evenson [32]. These cutpoints were recommended in a comparative validity study of accelerometer cutpoints [37]. Valid accelerometer data from 183 children were available (4 children were excluded because of not wearing the accelerometer or because of illness during the study week). Sedentary time, light intensity physical activity (LPA) and MVPA were calculated during recess and during the entire school day by using a school-specific time filter. A school day started when the first school bell rung, until the last school bell rung and was school specific. On Wednesdays the school day ends at noon. Only children who had data during the whole recess (20 minutes in school 1 and 2 and 15 minutes in school 3) of at least 1 baseline and 1 intervention recess were included in the analyses. Children were included in the analyses concerning the whole school day if they had at least 2 h 45 minutes of valid wear time per day of at least 1 baseline and 1 intervention day.

Based on the median of children’s average MVPA during usual recess, children were divided into two groups: most active and least active children (activity status).

### Feasibility questionnaire

After the intervention, all principals from the participating schools filled in a questionnaire about the feasibility of the intervention. Questions were scored on a 5-point Likert scale ranging from totally disagree (1) to totally agree (5). The outline of the feasibility questionnaire is shown in Table 1. Besides these questions, principals were also asked to report any difficulties/concerns and advantages of the intervention. The principals also reported recess times, available play and sports materials during recesses and the days on which physical education classes took place.

**Table 1:** Outline of the feasibility questionnaire

	**Totally disagree**	**Disagree**	**Sometimes agree/sometimes disagree**	**Agree**	**Totally agree**
	**(n)**	**(n)**	**(n)**	**(n)**	**(n)**
**Was it difficult as a principal, to halve the children at the playground during recess?**		1	1	1	
**Was it difficult for teachers to halve the children at the playground during recess?**		1		1	1
**Did the teachers support the intervention?**				3	
**Did children enjoy the intervention?**				3	
**Was this intervention useful for the children?**		1	1	1	
**Is it possible to use this intervention further in the future?**		2	1		

N = number of principals answering the question.

### Analyses

Rainy days were excluded from the analyses (n = 2). SPSS 20 was used to describe characteristics of the sample. Two-level (class - participants) linear regression analysis with random intercept and fixed slope was conducted in MLwiN 2.25 (a software package for fitting multilevel models in Windows) to examine the cross-sectional baseline association between play space and sedentary time, LPA and MVPA (controlled for age, sex, and recess duration). Three-level (class - participants – measurements (baseline or intervention)) linear regression analyses with random intercept and fixed slopes were conducted in MLwiN 2.25 to investigate intervention effects. The type of correlation structure was exchangeable. Intervention effects were examined on physical activity and sedentary time during recess and during the entire school day to test the activitystat hypothesis. Analyses concerning recess were controlled for age, sex, school, recess duration and recess period. Analyses concerning the entire school day were controlled for age, sex and school. Interaction effects with gender and activity status during recess were examined [38]. To test the interaction effect of gender and activity status, the cross-product terms of gender and intervention condition; and activity status and intervention condition were included in the multilevel linear regression models. When significant interactions between gender or activity status and intervention effects were found, separate models were fitted for boys and girls and for most and least active children. The IGLS estimation method in MLwiN was used to conduct the multilevel regression analyses. P-values ≤ 0.05 were considered as significant with exception for the interaction terms were it was set at p ≤ 0.1. Higher significance levels are used for interaction terms as they have less power [39]. An overview of the included variables in each model was provided as an Additional file 1.

## Results

### Descriptive results and baseline associations

The mean age of the participating children was 10.4 ± 0.9 years and 51.6% were boys. During usual recess, children engaged on average in 3.95 ± 2.37 (21.7%) minutes of sedentary time, 9.74 ± 2.18 (53.4%) minutes LPA and 4.55 ± 3.10 (25.0%) minutes of MVPA. The duration of the recesses ranged from 15 minutes to 20 minutes. On a usual school day, children spent 64.9% of the time being sedentary, 28.6% of the time they engaged in LPA and 6.5% in MVPA.

During usual recess, the available play space per child was 12.18 ± 4.19 m^2^; during intervention the available play space per child was 24.24 ± 8.51 m^2^ (Table 2).

**Table 2:** Descriptive characteristics of the recesses

	**School 1**		**School 2**		**School 3**		**Average of schools**	
**Variable**	**Baseline**	**Intervention**	**Baseline**	**Intervention**	**Baseline**	**Intervention**	**Baseline**	**Intervention**
	**mean**	**mean**	**mean**	**mean**	**mean**	**mean**	**mean ± SD**	**mean ± SD**
Duration (min)	20.0	20.0	20.0	20.0	15.0	15.0	18.2 ± 2.4	18.2 ± 2.4
Number of children	110	59	245	124	146	40	167 ± 70	84 ± 35
Playground area (m^2^)	972	972	4134	4314	1580	1580	2228.7 ± 1677.8	2228.7 ± 1677.8
Available play space per child (m^2^/child)	8.9	16.5	16.9	33.3	10.8	22.9	12.2 ± 4.2	24.2 ± 8.5
Duration of the school day	8h50 – 15h30		9h00-16h00		8h55 – 15h45			
Recess times								
Before noon	10h30-10h50		10h40-11h00		10h35 -10h50			
Lunch time recess	13h00 – 13h50		12h10 – 13h10		11h55 – 13h00			
Afternoon recess	/		14h50-15h10		14h40 – 14h55			
Available sports material during recess	Soccer goals/basketball hoops		Playground equipment (e.g. climbing frame)		Playground equipment (climbing frame, slide)			
	Box with play equipment		Box with play equipment		Box with play equipment			
	Hard surface area		Field with grass		Field with grass			
			Field with sand		Hard surface area			
			Hard surface area					
Physical education classes	4th grade: Tuesday and Friday		4th, 5th and 6th grade:		4th grade: Tuesday			
	5th grade: Tuesday and		Tuesday and Friday		5th grade: Monday			
	Thursday				6th grade: Friday			
	6th grade: Thursday and							
	Friday							

SD = standard deviation.

At baseline, the available play space was positively associated with minutes of MVPA during recess (β = 0.346 ± 0.066; p < 0.001) and negatively associated with minutes of sedentary time during recess (β = −0.188 ± 0.070; p = 0.008). The negative relation between available play space and minutes of LPA was only marginally significant (β = −0.162 ± 0.085; p = 0.055).

### Intervention effects during recess

Intervention effects of lowering playground density during recess are described in Table 3. Lowering playground density had a significant effect on lowering sedentary time (−0.58 min/recess; -3.21%/recess) and on increasing MVPA (+1.04 min/recess; +5.9%/recess) during recess. Sex was not a significant moderator of this relation.

**Table 3:** Intervention effects on physical activity and sedentary time during recess and the entire school day

		**During recess**^**a**^						**During school time**^**b**^		
		**Min SED (SD)**	**% SED (SD)**	**Min LPA (SD)**	**% LPA (SD)**	**Min MVPA (SD)**	**% MVPA (SD)**	**% SED (SD)**	**% LPA (SD)**	**% MVPA (SD)**
**Total sample**	Baseline	3.95 (2.37)	21.94 (13.13)	9.74 (2.18)	53.67 (10.95)	4.55 (3.10)	24.39 (15.60)	64.91 (6.44)	28.60 (5.69)	6.48 (2.68)
	Intervention	3.37 (2.42)	18.73 (13.57)	9.28 (2.23)	50.98 (10.58)	5.59 (3.14)	30.29 (15.76)	61.62 (9.54)	30.74 (6.90)	7.64 (4.57)
*χ*^2^		8.1**	6.3**	2.4	2.0	15.8***	13.5***	24.9***	19.5***	17.1***
**Boys**	Baseline	2.83 (1.88)	15.68 (10.53)	9.47 (2.29)	52.05 (12.07)	6.03 (3.16)	32.27 (15.58)	62.61 (6.76)	29.49 (6.34)	**7.90 (2.60)**
	Intervention	2.04 (1.32)	11.47 (7.90)	8.96 (2.22)	49.30 (10.94)	7.25 (2.94)	39.23 (13.69)	58.80 (9.95)	31.67 (7.20)	**9.53 (4.86)**
*χ*^2^		12.1***	9.3**	1.3	1.1	12.7***	11.1***	15.4**	9.4**	13.8***
**Girls**	Baseline	5.11 (2.27)	28.48 (12.41)	10.02 (2.04)	55.37 (9.43)	3.01 (2.14)	16.16 (10.60)	67.26 (5.15)	27.70 (4.81)	**5.03 (1.87)**
	Intervention	4.77 (2.54)	26.41 (14.11)	9.62 (2.21)	52.75 (9.95)	3.82 (2.27)	20.84 (11.81)	64.54 (8.19)	29.78 (6.48)	**5.68 (3.26)**
*χ*^2^		0.9	0.7	1.3	1.0	4.2*	3.5	9.4**	9.4**	4.1*
**Most active**	Baseline	**2.54 (1.59)**	**13.39 (8.02)**	9.46 (2.23)	50.27 (11.09)	**6.88 (2.56)**	**36.33 (12.48)**	**63.02 (6.28)**	**28.81 (5.79)**	**8.16 (2.32)**
	Intervention	**2.45 (1.71)**	**13.17 (9.33)**	9.23 (2.19)	48.94 (10.22)	**7.19 (2.88)**	**37.90 (13.90)**	**57.98 (9.11)**	**32.10 (6.66)**	**9.92 (4.61)**
*χ*^2^		**1.4**	**1.2**	0.5	0.2	**1.7**	**1.6**	31.1***	26.8***	16.4***
**Least active**	Baseline	**5.45 (2.13)**	**31.06 (11.27)**	10.04 (2.10)	57.30 (9.61)	**2.07 (0.91)**	**11.64 (4.60)**	**66.93 (6.01)**	**28.38 (5.61)**	**4.69 (1.71)**
	Intervention	**4.36 (2.68)**	**24.74 (14.87)**	9.34 (2.29)	53.18 (10.58)	**3.85 (2.41)**	**22.08 (13.40)**	**65.57 (8.41)**	**29.27 (6.89)**	**5.16 (2.97)**
*χ*^2^		9.8**	7.2**	3.5	2.7	30.0***	23.9***	**2.2**	**1.5**	**2.4**

***p < 0.001; **p < 0.01; *p < 0.05; SD = standard deviation; Min = minutes.

*χ*^2^ = chi square.

Bold = significant interaction effects (p < 0.1).

^a^analyses were controlled for: age, sex, school, recess duration and recess period; ^b^analyses were controlled for: age, sex and school.

Activity status of the children was a significant moderator of this relation for sedentary time (*χ*^2^ = 9.8; p < 0.001 for SEDmin and *χ*^2^ = 11.7; p < 0.001 for % SED) and MVPA (*χ*^2^ = 17.9; p < 0.001 for MVPAmin and *χ*^2^ = 19.9; p < 0.001 for % MVPA); in the least active children sedentary time significantly decreased (−1.09 min/recess; -6.32%/recess) and their MVPA increased during intervention (+1.78 min/recess; +10.44%/recess); whereas in the most active children no intervention effects were found during recess.

### Intervention effects during school time

Sedentary time significantly decreased (−3.29%/school day) and LPA and MVPA increased (+2.14% and +1.16%/school day) during school time when playground density was lowered. The moderating effect of sex was significant (*χ*^2^ = 3.5; p = 0.063) as boys’ MVPA during school time increased more during the intervention (+1.63%/school day) whereas the MVPA increase was lower in girls (+0.65%/school day). Also the activity status of the children during recess significantly moderated this relationship ((*χ*^2^ = 8.1; p = 0.003 for % SED and *χ*^2^ = 6.492; p = 0.011 for % LPA and *χ*^2^ = 5.2; p = 0.022 for % MVPA). For the most active children, sedentary time during the school time decreased during the intervention (−5.04%/school day) and LPA and MVPA increased during intervention (+3.29%/school day and +1.76%/school day respectively), whereas no intervention effects were found in the least active children during school time (Table 3).

### Feasibility

Two schools reported that the intervention was not very useful for their children, as they already had enough space to be physically active. The third school would implement this intervention only on rainy days, as children have insufficient space to play when it rains. All principals agreed that children enjoyed the intervention (n = 3) and that teachers supported the intervention (n = 3).

The following additional difficulties with lowering playground density during recess were reported by the school principals: more supervisors were needed (n = 2), there were incomplete lesson times before and after the recess (n = 2), nuisance from recess while other classes having lessons is disturbing (n = 1) and some children reported to the principal that they preferred playing together with children from other classes (n = 1) (Table 1).

## Discussion

Lowering playground density resulted in significant changes of sedentary time, LPA and MVPA during recess. Children engaged in less sedentary time, less light-intensity physical activity, and more moderate- to vigorous-intensity physical activity during split recesses. These changes were comparable to the changes in a similar intervention that was conducted in preschoolers [26]. However, these positive changes were rather small compared to other recess interventions aiming to increase physical activity [21] (e.g. Verstraete et al. found an increase of 13% in MVPA by providing game equipment during recess while MVPA decreased in the control group [20]). This can be attributable to the short duration of the recesses in which the playground density was lowered (15–20 minutes) and the short period of the intervention (one week) compared to other studies were interventions were conducted during 4 weeks [40] or 3 months [41]. It might be possible that children had insufficient time to adapt their play behavior to the increased available space. Another possible explanation for the small effects is the fact that the available area per child was already very reasonable and quite high; therefore it might be possible that children already had enough space to move around as they wished. Or alternatively the increase in play space may have been not large enough to alter children’s PA or sedentary time during recess. In a study by Harten et al. children were more active in medium (71.6 - 86.8 m^2^ per child) and large (130.4 – 157.9 m^2^ per child) play areas compared to small play areas (13.0 – 15.8 m^2^ per child); these areas were much larger than the intervention space in the present study [25].

Larger effects could possibly be found if a multicomponent intervention was used instead of only focusing on the increased available space as many intervention studies confirmed the positive effects of game equipment [20,42] and playground markings [40,43,44] on children’s physical activity levels during recess at elementary school. This intervention also might be more effective in schools with smaller playgrounds (e.g. inner-city schools): participating schools in this study already had large playgrounds compared to schools in other studies [24]. Also, implementing this intervention during lunch break could increase the effectiveness as lunch recess lasts longer than other recesses (± 1 hour) [20].

Similar to other studies we found that boys engaged in less sedentary time and more MVPA during recess compared to girls [24,45]. These differences remained significant during split recesses. Blatchford et al. showed that boys in primary school were more involved in ball games (e.g. throwing and catching, soccer, basketball and other derived games), whereas girls were more engaged in sedentary play, conversation, and rope skipping [46]; which explains the differences in physical activity levels during recesses in boys and girls. On the contrary, Mota et al. found in a small study sample that girls were more engaged in MVPA during recess compared to boys [47]; the majority of boys spent much of their playtime in sedentary play such as swapping game cards or simply talking; whereas girls continued participation in traditional, apparently more active, playground games.

The intervention effects during recess were similar in boys and girls. This was contrary to what was expected based on the findings of Harten et al. [25] who found that play space was a more important variable in determining the free play activity of boys compared to girls. The difference between the current study and the study of Harten et al. can be explained by the fact that Harten al. investigated the effect of change in play area by using markers to define out-of-bound areas, whereas in the current study, the area of the playground remained the same during the intervention, but only the number of children sharing the playground decreased. By decreasing the number of children sharing the playground, children have better access to the available play equipment (e.g. jumping ropes, balls,..), as this equipment need to be shared with less children. This may have led to the fact that intervention effects were similar in boys and girls as both had easier access to their favorable play equipment. However during the entire school day, lowering playground density was effective in increasing boys’ MVPA but not girls’ MVPA, so boys did not compensate more MVPA during recess at another moment during the school day. Girls who engaged in more MVPA during recess, compensated for this increase by decreasing their MVPA during another moment during the school day (e.g. being less active during physical education class). The finding that physical activity interventions were more effective in boys is consistent with the results of an environmental intervention study in the US, aiming at increasing physical activity at schools [48]. Therefore, tailored physical activity interventions are necessary; aiming at increasing girls physical activity during the school day.

The intervention was more effective in the least active children, compared to the most active children during recess. Children that were least active during usual recess engaged in less sedentary time and more MVPA during the split recess, whereas these effects were not significant in the most active children. Apparently the most active children want to be active during recess, regardless of the available space, probably because they are more autonomously motivated towards PA [27]; whereas less active children probably experience more opportunities to be physically active due to the increase in play space when recess was split. By increasing the play space during recess, it is easier to participate in active games and make use of game equipment and playground markings during recess which results in higher physical activity levels during recess in least active children. The positive intervention effect that was found during recess in least active children was not found during the entire school day. It seems that the least active children compensate less sedentary time and more MVPA during recess with more sedentary time and less MVPA at another moment during the school day [28]. Consequently, strategies are needed to maintain the increase of MVPA in least active children during the entire day, by implementing multi-component interventions to increase physical activity during the school day. During the entire school day, most active children engaged in less sedentary time, more LPA and more MVPA during the intervention condition, whereas no significant differences were found during recess. These effects are probably not due to the playground intervention, as this intervention was not effective during recess in most active children but can be due to other things (e.g. more activity during lunch recess on the intervention days, physical education during intervention days,…). Therefore, intervention effects during the entire school day need cautious interpretation.

Although this intervention was free of costs, did not require any adaptations of the school facilities and did not require teacher trainings, the school principals reported some difficulties when implementing the intervention. The need for more supervisors, the incomplete lesson times before and after recess (as lesson times were split up because recess started earlier or later as usual), nuisance from recess for other classes and the absence of friends of children from other classes that did not have recess the same time were some of the difficulties reported in the questionnaire. Also the low response rate of the schools (30.0%) could be explained by the low perceived feasibility of the intervention by the school principals as those principals did not find it feasible to implement the intervention. Due to these reported problems, none of the participating principals fully agreed that it is possible to use this intervention further in the future. However, one school principal would implement the intervention in the future on rainy days. As elementary schools in Flanders often have lack of place inside for recess when it rains, lowering the playground density can be a valuable solution to solve this problem. Van Cauwenberghe et al. conducted a comparable study in preschoolers [26]. In preschools, principals reported a higher feasibility and this is possibly due to the fact that preschools have less strict timetables. A possible solution for some of these difficulties is the implementation of the intervention before and after lunch time instead of implementing this intervention during the shorter recess before noon or during the afternoon. In Belgium, children have lunch when recess during the noon starts (during 20–30 minutes); after lunch time children have noon recess outside. Half of the children could start the noon recess with lunch time, while the other half of the children could have recess outside before lunch time. By doing so, lessons will not be disturbed by the nuisance of the recess, lesson times will remain complete and positive intervention effects can be maintained or even enlarged as recess during noon usually lasts longer than recess before noon or in the afternoon. However, it might be possible that some children who go home during lunch recess (16.8% of Flemish school children, unpublished data) miss the positive intervention effects of decreasing playground density. Also the need for more supervisors cannot be solved by implementing the intervention during lunch time.

Despite these difficulties, all principals agreed that children enjoyed the intervention and positive intervention effects were found, so it might be worth to further investigate the effects of lowering playground density during lunch time.

The small number of children and schools included in the study due to the low perceived feasibility; the short intervention period were limitations of this study. As accelerometers were handed out on Monday morning before the first recess and collected on Friday afternoon after the last recess, Mondays and Fridays were measured incomplete which made our data unsuitable to investigate the intervention effects during the entire day (including data before and after school time). This also forms a limitation of the study. Baseline characteristics concerning play equipment and type of play space differed between the schools, however as a within-subject design was used, the influence of these different baseline characteristics was minimal. However it is possible that a larger increase of PA was measured in the schools with more playground equipment, as this equipment became more available for the children during intervention. The use of accelerometers as objective measurement tools for physical activity and sedentary time, the high external validity due to the within subjects design and the measurements during the whole school day were strengths of the study.

## Conclusions

The present study showed that lowering playground density can be an effective intervention for decreasing children’s sedentary time and increasing their physical activity levels during recess. Although positive intervention effects were small, decreasing playground density could be very valuable and effective in a larger multi-component intervention in schools to increase physical activity and to decrease sedentary time. Further research should focus on the effect of this intervention during lunch time in schools with denser playgrounds, as this can enlarge the intervention effects and forms a solution for a lot of the reported difficulties by the principals.

## Abbreviations

LPA: Light intensity physical activity; MVPA: Moderate-to vigorous-intensity physical activity.

## Competing interests

The authors declare that they have no competing interests.

## Authors’ contributions

SDH conducted the statistical analyses and drafted the manuscript. GC, IDB, and DVD participated in the interpretation of the data, helped to draft the manuscript and revised the manuscript for important intellectual content. All authors read and approved the final manuscript.

## Pre-publication history

The pre-publication history for this paper can be accessed here:

http://www.biomedcentral.com/1471-2458/13/1154/prepub

## Supplementary Material

Additional file 1 Intervention effects during recess for the whole sample. Click here for file
