# Supplementary material for: Effectiveness and feasibility of lowering playground density during recess to promote physical activity and decrease sedentary time at primary school
Source: BMC Public Health. 2013 Dec 10;13:1154. doi: 10.1186/1471-2458-13-1154 (PMC3878886; doi:10.1186/1471-2458-13-1154)
Supplement: Additional file 1 — Intervention effects during recess for the whole sample. [file 1471-2458-13-1154-S1.docx]

**Additional file 1. Intervention effects during recess for the whole sample**

|  | During recess | | | | | | During school day | | |
| --- | --- | --- | --- | --- | --- | --- | --- | --- | --- |
|  | *Min SED*  *β±SE* | % SED  *β±SE* | Min LPA  *β±SE* | % LPA  *β±SE* | Min MVPA  *β±SE* | %MVPA  *β±SE* | % SED  *β±SE* | %LPA  *β±SE* | %MVPA  *β±SE* |
| FIXED PART |  |  |  |  |  |  |  |  |  |
| Covariates |  |  |  |  |  |  |  |  |  |
| Sex (ref = boy) | 2.36±0.24 | **13.02**±1.32 |  |  |  |  |  |  |  |
| School (ref = school 1)  School 2  School 3 | -1.11±0.47  -0.96±0.35 |  |  |  |  |  |  |  |  |
| Age | 0.24±0.14 |  |  |  |  |  |  |  |  |
| Recess duration | 0.00±0.00 |  |  |  |  |  |  |  |  |
| Recess period (ref=before noon) | -0.096±0.35 |  |  |  |  |  |  |  |  |
| Predictor |  |  |  |  |  |  |  |  |  |
| Condition (ref = baseline) | -0.60±0.21 |  |  |  |  |  |  |  |  |
| RANDOM PART full model | σ^2^±SE |  |  |  |  |  |  |  |  |
| Class level variance | 0.00±0.00 |  |  |  |  |  |  |  |  |
| Pupil level variance | 1.29±0.30 |  |  |  |  |  |  |  |  |
| Measurement level variance | 2.57±0.27 |  |  |  |  |  |  |  |  |

Β = multilevel linear regression coefficient; SE = standard error;
